# Lessons Learned From the Implementation of a School-Based Sexual Health Education Program for Adolescent Girls in Cape Town, South Africa

**DOI:** 10.9745/GHSP-D-23-00026

**Published:** 2023-12-22

**Authors:** Carey Pike, Chelsea Coakley, Devyn Lee, Derek Daniels, Nadia Ahmed, Miriam Hartmann, Nancy Padian, Linda-Gail Bekker

**Affiliations:** a Faculty of Health Sciences, Department of Medicine, Desmond Tutu HIV Centre, University of Cape Town, Cape Town, South Africa.; b Independent Research Consultant, Adolescent Health and Development, Cape Town, South Africa.; c Grassroot Soccer, Cape Town, Cape Town, South Africa.; d Mortimer Market Centre, Central North West London NHS Trust, London, United Kingdom.; e Karolinska Institutet, Department of Global Public Health, Stockholm, Sweden.; f University of California Berkeley, Berkeley, CA, USA.

## Abstract

Comprehensive sexual education (CSE) is an important approach for health promotion dissemination in schools, particularly when delivered in participatory ways that are sport based, peer or near-peer led, and include concepts of gender and power dynamics. Sufficient attendance at CSE sessions is critical to ensure content exposure and build potential for impact. This article details implementation lessons from the delivery of an after-school, near-peer-led CSE program called SKILLZ during a cluster randomized controlled trial among 40 secondary schools in Cape Town, South Africa. SKILLZ is an evidence-based program previously implemented in similar contexts. Attendance in this study was suboptimal (less than 50%) despite qualitative indications of high acceptability and extensive efforts to adapt implementation and boost attendance. Qualitative and quantitative data gathered during anonymous surveys, in-depth interviews, and focus group discussions with participants with high and low attendance records, near-peer coaches, and school staff from both cohorts indicated that poor attendance could be attributed to several factors. Program adaptations, such as delivering difficult-to-access health services before participants attended the SKILLZ program rather than after, may have reduced the incentive for young people to attend the program. Practical barriers to attendance were identified, including security concerns, competing priorities, and unfamiliarity with after-school programming culture in this setting. Community engagement and acceptability of sexual education programs for adolescents alone may be insufficient to ensure program uptake and engagement.

## Abstract (precis)

Community engagement and acceptability of sexual education programs for adolescents alone are insufficient to ensure program uptake and engagement.

## Teaser

**Key Findings:**

- Attendance at an evidence-based, near-peer-led comprehensive sexual education program for female adolescents in South African secondary schools was suboptimal, even after extensive community engagement and modifying the delivery of the program based on the school's preference.
- Participants reported high interest and acceptability, but this did not translate to consistent attendance because of barriers, including participants' security concerns and competing priorities on their time.
- Providing adolescent-friendly health care screenings early as part of the study may have influenced learners' desire to later attend the program intervention sessions.

**Key Implications:**

- National stakeholders in South Africa should consider new ways to address barriers to after-school programming, without which adolescent learners may be deprived of access to extracurricular activities. In-school programming, although effective, is not a sustainable strategy to overcome these barriers because if it is overused, it risks congesting the school day and interfering with core educational activities.
- Program managers should promote clear roles and responsibilities among near-peer educators and limit research facilitation responsibilities.

## BACKGROUND

Adolescents show disproportionately poor sexual and reproductive health (SRH) outcomes compared to other age groups, with high rates of unintended pregnancy and sexually transmitted infections (STIs), including HIV infections.^1^^–^^3^ School-based, comprehensive sexuality education (CSE) is an effective strategy to reduce sexual risk factors that may drive these outcomes by helping HIV and SRH knowledge, enhancing self-efficacy, and providing practical tools to better navigate safe sex practises.^4^^,^^5^

Schools can provide a safe, equipped, and conducive educational environment in which to reach adolescents through both classroom-based and after-school extramural activities.^3^ In South Africa, school attendance is compulsory until the age of 15 years, and the majority of adolescent girls (96.4% in the Western Cape Province) are enrolled in secondary school.^6^ Half of South Africans aged 15–23 years report having sex before age 18 years (whether consensual or not). Thus, schools provide an appropriate platform where adolescents can participate in SRH interventions that improve SRH knowledge, SRH risk management, and self-efficacy before and after sexual debut, maximizing the potential to improve SRH outcomes.^7^ The capacity for the impact of SRH programs is linked to content exposure, necessitating high program attendance. Attendance can be influenced by program acceptability, feasibility, and variability in implementation.^8^^,^^9^

Schools provide an appropriate platform where adolescents can participate in SRH interventions that improve knowledge, risk management, and self-efficacy before and after sexual debut, maximizing the potential to improve SRH outcomes.

We report on implementation lessons learned from an evidence-based sexual health education program in Cape Town, South Africa, called SKILLZ for Girls (SKILLZ), which showed poor program attendance despite high acceptability and intervention support.

## THE SKILLZ FOR GIRLS INTERVENTION

SKILLZ is an evidence-based, near-peer-led CSE program that provides a girl-centered and participatory sports-based curriculum informed by social cognitive theory.^10^ The program was delivered during a cluster randomized controlled trial among female secondary school learners across 40 public schools in low-income, high-density, peri-urban locations in Cape Town, South Africa. Girl-centered programming was adopted in response to the disproportionately high SRH burden among adolescent girls in South Africa, including increased risk of HIV infection, higher prevalence of STIs, and unintended pregnancies.^11^^–^^16^ Although there is demand from adolescent girls who are primarily educated in coeducational environments to have a girls-only space to talk about SRH issues, the impact of SKILLZ may be bolstered by the implementation of parallel programming for boys.^8^

SKILLZ was developed in South Africa and has since been adapted and delivered to adolescent girls in other sub-Saharan African countries within similar low socioeconomic, peri-urban settings, with high rates of attendance (more than 90%) and had a modest impact on sociobehavioral indicators of gender norms, self-esteem, and sexual health knowledge.^17^^,^^18^ In this study, the curriculum was adapted to complement the national South African CSE program, which is a teacher-led, in-school, mandatory curriculum on life skills and SRH issues, with an emphasis on the consequences of early pregnancy and the importance of schooling. The SKILLZ curriculum focused purely on SRH lessons and went further to include gender and power relations, STIs, healthy relationships, and positive self-concept. These are all topics that have been previously shown to increase the efficacy of CSE interventions in reducing unintended pregnancy and HIV incidence.^5^^,^^19^ In keeping with the original SKILLZ program, sessions lasted 45–60 minutes and were held weekly during the school term, excluding exam periods and school holidays. Overall, the program delivered 10 sessions over 2 school terms (approximately 6 months). Participants who attended at least 7 of 10 sessions were considered to have met the threshold required to “graduate,” which assumed sufficient exposure to a predetermined degree of content to result in behavioral or attitudinal change.^8^

SRH knowledge is best promoted when teachers are comfortable with the material, programs are peer or near-peer led, and a strong facilitator-learner relationship is established.^20^^–^^22^ Yet CSE delivery in South Africa is challenged by a rarity of trusting, nonjudgmental relationships between teachers and students, the influence of conservative teacher beliefs on SRH content, and the lack of appropriate training opportunities for teachers.^20^^–^^22^ Therefore, SKILLZ sessions were facilitated by trained, near-peer “caring coaches” (aged 20–30 years) over a 6-month period (2 school terms), with a ratio of 1 coach per 25 participants. A near-peer coach was defined as a nonprofessional group facilitator and role model of a similar age, gender, and social group to the learner recruited from their community. Recruitment criteria for near-peer coaches were individuals who identified as female, were aged 20–30 years (median age recruited was 25 years), completed secondary school, and resided in the study area. When recruiting coaches, these criteria were equally considered. Recent evidence (2017–2022) supports the use of near-peer and peer-mentorship models to build adolescent and young people's assets and pre-behavioral and behavioral outcomes when paired with intentional community engagement and health systems interventions.^23^^–^^26^ Coaches received a 1-week training program and thereafter weekly professional development sessions that included sessions on research, facilitation, and the development of professional “soft skills.” Coaches were mentored and supported by a dedicated master coach who had worked a minimum of 3 years as a coach in other SKILLZ programs in a similar setting. This approach is consistent with the original SKILLZ program. However, a key difference in this study compared to previous SKILLZ programs was that coaches were given additional research responsibilities, such as assisting with data collection, recruiting participants, and facilitating attendance reminders. Coaches were remunerated in line with their dual facilitator and research roles, with salaries greater than those provided in previous editions of SKILLZ.

The primary aim of the study, the results of which are reported elsewhere,^27^ was to evaluate the SKILLZ program's impact on SRH indicators, including prevalence of HIV, STIs, and pregnancy, as well as self-reported sociobehavioral measures of social support, self-concept, and gender norms. The study hypothesized that exposure to educational content and an opportunity for coach-participant relationship building would facilitate improvements in SRH and sociobehavioral indicators, with a linear correlation between attendance and program impact. During the adaption of SKILLZ for this study, factors at the individual, relationship, and community (school) levels were considered with the potential to modify the intervention effect on the outcomes. Mediating outcomes were hypothesized to include perceived social support and norms, degree of empowerment, self-concept and self-efficacy, trust in the health care system, the schools, and the near-peer “coaches,” and perceived stigma. Based on qualitative and quantitative data gathered from anonymous surveys, in-depth interviews, and focus group discussions with high- and low-attending participants, coaches, and school staff, factors considered to influence the fidelity of program implementation are reported here, which include participant, school, and coach receptivity to and understanding of SKILLZ, perceived appropriateness and relevancy of SKILLZ, as well as potential barriers and enablers to attendance.

### Study Sites

Between 2018 and 2019, all 42 secondary schools located in the study area, a single health subdistrict within the Western Cape Province, were randomized to be a control school (n=20), intervention school (n=20), or nonparticipating school (n=2). For logistical feasibility, study implementation was split over 2 years, with 20 schools (10 intervention, 10 control) engaged in 2018 (cohort 1) and 20 schools (10 intervention, 10 control) engaged in 2019 (cohort 2). Eligible learners within each school were required to be female, not pregnant, and in school grades 8–10 (approximately aged 13–17 years). Pregnancy testing was included, as pregnancy incidence was a primary study outcome. Pregnancy tests were conducted in private by trained adolescent-friendly health care professionals, and all participants who received a positive pregnancy test were counseled and referred for further care if needed. The program was advertised by poster and in-class promotion by study staff. Between 70 and 100 learners per school self-selected to participate. Participants were followed prospectively for a period of 8 months, with a total sample size target of 2,800.

School and community engagement before study commencement was extensive, involving stakeholder consultations at the provincial, district, and individual school levels. Each school principal nominated a “teacher champion” who acted as the school liaison between the implementing team, school staff, and participants. At that time, the implementing research team had an established presence in all participating schools through a larger national women and girls program that included the delivery of SRH education and services in schools. A Youth Community Advisory Committee affiliated with the study site that had advised on several studies and clinical trials was consulted during the protocol development process. As part of the curriculum development process, a group of adolescent learners from participating schools were engaged in pilot SKILLZ sessions. Their feedback was incorporated into the curriculum design, notably changing the sport component of SKILLZ from solely soccer (football) to include other activities, such as yoga and stretching.

Intervention schools shared similar learner demographic profiles, with no significant differences in participant characteristics between control and intervention schools or between cohorts 1 and 2. Thirty-eight schools completed the study (18 in cohort 1 with 9 intervention and 9 control schools; 20 in cohort 2 with 9 intervention and 11 control schools). Two schools withdrew mid-implementation due to a change in local policy that was interpreted as disallowing external providers to provide health care services, such as HIV and pregnancy testing, during study visits on school premises. One control school in cohort 1 requested to delay participation due to building renovations on the school premises and, as such, was included in cohort 2.

Of 2,791 participants enrolled, 1,344 (48.15%) participants were included in the intervention arm and 1,447 (51.84%) participants were included in the control arm; 44.39% (1,239) participated in cohort 1 and 55.61% (1,552) participated in cohort 2.^27^ Control and intervention participants were required to attend 2 study visits (independent of SKILLZ program sessions) at baseline and 8 months, where demographic, clinical (including HIV, STI, and pregnancy testing), and sexual risk behavior data were collected. Although 73.62% (2,119) of all participants attended both study visits, 17.52% (489) were lost to follow-up and uncontactable across multiple follow-up attempts. Despite high study visit attendance among participants, their attendance at SKILLZ was low across both cohorts, and overall, 41.14% of participants did not participate in any session (44.43% in cohort 1; 38.63% in cohort 2).

### Ethical Approval

Ethical approval was granted by the Human Research Ethics Committee at the University of Cape Town (REC 138/2018). The trial was registered with BMC Trials (ISRCTN77395422).

## SKILLZ ATTENDANCE

In cohort 1, 9 schools received the intervention program, with sessions delivered after school as intended. Mechanisms to support attendance, identified through the literature and the study team's experience of SKILLZ delivery, included providing refreshments, offering optional free transport home after sessions, and engaging a school-appointed “teacher champion” at each school (Table).^28^ Qualitative focus group discussions with participants, interviews with school staff, and self-administrated post-intervention surveys with participants found unanimously that SKILLZ was perceived as highly acceptable and responsive to gaps in existing SRH programming and in learner knowledge. School staff reported appreciating that the program facilitated SRH conversations with learners, as the SRH programs were considered scarce, and teachers did not always feel equipped to facilitate these discussions in class. In cohort 1, 78.34% (340/434) of participants rated their interest in the sessions as 8 or higher on a scale of 10 and further indicated that they valued the intentional design of the intervention, including the girls-only peer group, sport-based participatory learning medium, and safe spaces created by coaches.

**TABLE.** Comparison of Mechanisms of Support Offered to Schools Receiving SKILLZ in 2 Cohorts

Cohort 1	Cohort 2
Sent session reminders to school Provided refreshments Provided transport Engaged teacher champion Provided certificate upon graduation (communicated in advance to participants)	Used preferential program delivery during school class time, during lunchtime, or after school Sent session reminders to school and directly to participants via WhatsApp Provided refreshments Provided transport Engaged teacher champion Provided certificate and medal provided upon graduation (communicated in advance to participants)

Despite this positive feedback, cohort 1 showed a mean attendance rate of 44.20% (standard error [SE]: 5.22%; 95% confidence interval [CI]=33.18%, 55.22%) and only 27.79% graduated from the program. Safety concerns linked to high levels of community unrest, violence, and gang activity were commonly reported as a reason for non- or infrequent attendance. To mitigate some of these known concerns, private transportation was offered. However, this option was not always sufficient if the driver could not take the learner directly to their place of residence. This differs from other similar settings in Cape Town where SKILLZ has been delivered and where private transportation arrangements were able to mitigate safety concerns and allow for high attendance. Participants also described competing priorities (e.g., homework and household responsibilities, such as caring for a younger sibling) as a common barrier to attendance after school. Low attendance at other after-school programs in the participating schools was found to be widespread, with little to no culture supporting attendance. School records showed that although 7 of the 9 cohort 1 schools had existing after-school programs other than SKILLZ, only 2 of those schools reported high attendance at after-school programs. Notably, the culture of after-school programming has not previously presented such significant challenges regarding low and nonattendance when delivered in similar settings.

Despite participants having positive feedback about the program sessions, cohort 1 had a mean attendance rate of 44%.

An assessment conducted after the conclusion of cohort 1 explored alternative delivery models, including in-school sessions (either during class periods or lunchtimes), weekends, and school holidays. Although the latter options were found unequivocally unfavorable to participants, in-school delivery during free class periods or lunchtime periods was perceived as acceptable and led to more than 90% in attendance when piloted in cohort 1 schools. These findings motivated an adaption to allow cohort 2 schools to choose between in-school or after-school session delivery based on the school's preference, alongside the introduction of other additional attendance support mechanisms (Table).

Nine new intervention schools were engaged in cohort 2, of which 5 schools opted to receive an in-school program (3 during class time and 2 during lunch time) and 4 selected after-school delivery. Compared to cohort 1, cohort 2 intervention schools did not show a significant improvement in attendance, with a mean attendance rate of 47.17% (SE: 4.90%; 95% CI=36.81%, 57.52%) and a mean difference in attendance rate of 2.97% (*P*=.684).^27^ However, schools that opted for in-school delivery showed significantly higher rates of graduation (50.24%) compared to after-school delivery (18.69%, *P*<.05), as well as an observable but not significant improvement in graduation compared to cohort 1 (27.79%). Although SKILLZ coaches perceived in-school delivery as more effective, this option did risk overburdening learners within the school day, especially for those who participate in multiple programs.

Overall, the school-informed modifications made to support SKILLZ delivery in cohort 2 did not elevate attendance to levels comparable with other SKILLZ programs. A key difference in this study compared to other SKILLZ programs was the provision of a health care screening visit at the beginning of the program before any SKILLZ sessions were delivered. In other SKILLZ programs, health care screenings were offered midway or toward the end of the intervention as a means of incentivizing attendance and reinforcing health promotion messaging. Health care access for adolescents in South Africa has been well described as suboptimal, limited by inconvenient clinic opening times and adolescent perceptions of unfriendly health care staff.^29^ This study provided adolescent-friendly health services (as per the World Health Organization's standards) directly on the school premises at baseline and 8 months. Health care service access may have been viewed as an incentive to join the study in this setting. The early provision of the health care screenings (overall, 73.62% attended both health care screenings) may have influenced the desire to attend subsequent intervention sessions (only 58.86% attended at least 1 SKILLZ session).

School-informed modifications made to support SKILLZ delivery in cohort 2 did not elevate attendance to levels comparable with other SKILLZ programs.

An SRH program for adolescent girls in Zambia showed a similarly low attendance rate, where only 30% of participants attended more than half of the sessions.^30^ This was attributed to the high vulnerability of the participants and suggested that the very social and economic factors that made them vulnerable to poor SRH outcomes also prevented them from participating consistently in SRH programs. This supports the argument that SRH interventions need to be delivered as part of multilevel combination programs that layer educational components with health care services and social protection measures, for example, in the model proposed in the multicountry DREAMS (Determined, Resilient, Empowered, AIDS-free, Mentored and Safe) intervention that focused on HIV prevention in adolescent girls and young women.^31^

## LESSONS LEARNED

We learned the following key lessons from our experience in sexual health program delivery in a low-resource South African setting.

### High Acceptability Is Not Sufficient to Overcome Logistical and Structural Barriers

A systematic review of school-based skills development interventions showed that intervention success is commonly associated with the acceptability of programs among participants and variation in implementation.^32^ However, high program acceptability of SKILLZ did not translate to consistent attendance or practical implementation support from school staff, with a wide variety of implementation barriers and enablers observed across schools. These barriers reduced the feasibility of the program overall and likely undermined its potential to positively impact SRH outcomes. A further consideration is that participants may have found the program to be acceptable but insufficiently relevant to their perceived needs. However, the high attendance rates during in-school sessions (over lunch times or free class periods) indicate a willingness to participate if practical barriers to attendance are removed.

### Schools Differ in Ability to Accommodate After-School External Programming

Adjusting the timing of program delivery to the school's preference may benefit schools that can accommodate in-school delivery. However, those schools that select after-school models may need additional support to introduce, build, and maintain an after-school culture. Although they are considered homologous units in cluster randomized trials, schools in the same geographic and demographic settings may still vary markedly in their ability to effectively participate in research and after-school activities.

### SRH Service Access May Have Incentivized Visit Attendance

Although attendance at the intervention program sessions was low, 73.62% of all control and intervention participants attended both study visits where adolescent-friendly SRH services were offered. HIV, STI, and unintended pregnancy are all highly prevalent issues in the South African context, with adolescent girls and young women at particularly high risk and often targeted for intervention. Barriers to adolescent SRH services from public clinic facilities are well described. Although school-based health services have been long supported in South African national policy, their implementation has been stalled for more than a decade.^32^^,^^33^ As such, the high demand for these services is not surprising and may explain why study visit attendance was higher than SKILLZ program attendance.

### Near-Peer Educators Should Have Clear Roles and Responsibilities

An effective participant-coach relationship has been reported in previous research on SKILLZ as being important for intervention acceptability.^17^^,^^18^ In this study, coaches had additional research responsibilities, a departure from other SKILLZ programs that are normally not delivered in conjunction with research studies. The coaches showed varied capacity to adapt systematically to implementation challenges and a propensity to conflate research and programmatic priorities. As a significant program resource, it is recommended to clearly delineate between program facilitation and research responsibilities.

## CONCLUSION

Despite high acceptability among stakeholders, extensive community engagement before implementation, and the introduction of additional support mechanisms, this evidence-based SRH program showed suboptimal attendance that likely compromised its potential to impact SRH outcomes and the opportunity for that potential to be evaluated. Poor attendance at least partially occurred due to practical barriers to attendance at the individual, school, and society levels and included security concerns, competing constraints on participant time, and the lack of an after-school programming culture. Under such circumstances, the use of other evidence-based methods (e.g., combination prevention programs) or practical adaptions to move aspects of programs in-school or online may be considered.

## Notes

**Peer Reviewed**

**First published online:** December 15, 2023.

## References

[1] Patton GC, Sawyer SM, Santelli JS, et al. Our future: a Lancet commission on adolescent health and wellbeing. Lancet. 2016;387(10036):2423–2478. 10.1016/S0140-6736(16)00579-1. 27174304 PMC5832967

[2] Naidoo S, Wand H, Abbai N, Ramjee G. High prevalence and incidence of sexually transmitted infections among women living in Kwazulu-Natal, South Africa. AIDS Res Ther. 2014;11(1):31. 10.1186/1742-6405-11-31. 25243015 PMC4168991

[3] Adolescent pregnancy. World Health Organization. June 2, 2023. Accessed December 4, 2023. https://www.who.int/news-room/fact-sheets/detail/adolescent-pregnancy

[4] Gallant M, Maticka-Tyndale E. School-based HIV prevention programmes for African youth. Soc Sci Med. 2004;58(7):1337–1351. 10.1016/S0277-9536(03)00331-9. 14759680

[5] Fonner VA, Armstrong KS, Kennedy CE, O'Reilly KR, Sweat MD. School based sex education and HIV prevention in low- and middle-income countries: a systematic review and meta-analysis. PLoS One. 2014;9(3):e89692. 10.1371/journal.pone.0089692. 24594648 PMC3942389

[6] Richter L, Mabaso M, Ramjith J, Norris SA. Early sexual debut: voluntary or coerced? Evidence from longitudinal data in South Africa – the Birth to Twenty Plus study. S Afr Med J. 2015;105(4):204. 10.7196/SAMJ.8925. 26294851

[7] Eaton L, Flisher AJ, Aarø LE. Unsafe sexual behaviour in South African youth. Soc Sci Med. 2003;56(1):149–165. 10.1016/S0277-9536(02)00017-5. 12435558

[8] Hershow R, Gannett K, Merrill J, et al. Using soccer to build confidence and increase HCT uptake among adolescent girls: a mixed-methods study of an HIV prevention programme in South Africa. Sport Soc. 2015;18(8):1009–1022. 10.1080/17430437.2014.997586. 26997967 PMC4795818

[9] Craig P, Dieppe P, Macintyre S, Michie S, Nazareth I, Petticrew M; Medical Research Council Guidance. Developing and evaluating complex interventions: the new Medical Research Council guidance. BMJ. 2008;337:a1655. 10.1136/bmj.a1655. 18824488 PMC2769032

[10] Bandura A. Social cognitive theory of mass communication. Media Psychol. 2001;3(3):265–299. 10.1207/S1532785XMEP0303_03

[11] Johnson LF, Lewis DA. The effect of genital tract infections on HIV-1 shedding in the genital tract: a systematic review and meta-analysis. Sex Transm Dis. 2008;35(11):946–959. 10.1097/OLQ.0b013e3181812d15. 18685546

[12] Naidoo S, Wand H, Abbai N, Ramjee G. High prevalence and incidence of sexually transmitted infections among women living in Kwazulu-Natal, South Africa. AIDS Res Ther. 2014;11(1):31. 10.1186/1742-6405-11-31. 25243015 PMC4168991

[13] Gill K, Celum CL, Breen G, et al. High prevalence and incidence of curable STIs among young women initiating PrEP in a township in South Africa. Presented at: 23rd ISSTDR: STI & HIV 2019 World Congress; July 14–17, 2019; Vancouver, Canada. Abstract P432. 10.1136/sextrans-2019-sti.518

[14] Delany-Moretlwe S, Mgodi N, Bekker LG, et al. High curable STI prevalence and incidence among young African women in HPTN 082. Presented at: CROI 2019; March 4–7, 2019; Seattle, WA.

[15] Statistics South Africa. *Demographic Profile of Adolescents in South Africa*. Report Number 03-00-10. Statistics South Africa; 2018. Accessed November 1, 2023. http://www.statssa.gov.za/publications/Report%2003-00-10/Report%2003-00-102016.pdf

[16] Vollmer LR, van der Spuy ZM. Contraception usage and timing of pregnancy among pregnant teenagers in Cape Town, South Africa. Int J Gynaecol Obstet. 2016;133(3):334–337. 10.1016/j.ijgo.2015.10.011. 26895740

[17] Sanders B, Barkley C, Das M, Cooper A. Changing the game for girls: results from a longitudinal study of a soccer based HIV and SGBV prevention programme for adolescent girls in South Africa. Presented at: IAS 2017; July 23–26; Paris, France.

[18] Merrill KG, Merrill JC, Hershow RB, et al. Linking at-risk South African girls to sexual violence and reproductive health services: a mixed-methods assessment of a soccer-based HIV prevention program and pilot SMS campaign. Eval Program Plann. 2018;70:12–24. 10.1016/j.evalprogplan.2018.04.010. 29890449 PMC6613633

[19] Haberland NA. The case for addressing gender and power in sexuality and HIV education: a comprehensive review of evaluation studies. Int Perspect Sex Reprod Health. 2015;41(1):31–42. 10.1363/4103115. 25856235

[20] Visser MJ. Life skills training as HIV/AIDS preventive strategy in secondary schools: evaluation of a large-scale implementation process. SAHARA J. 2005;2(1):203–216. 10.1080/17290376.2005.9724843. 17601024

[21] Francis DA, DePalma R. ‘You need to have some guts to teach’: teacher preparation and characteristics for the teaching of sexuality and HIV/AIDS education in South African schools. SAHARA J. 2015;12(1):30–38. 10.1080/17290376.2015.1085892. 26365812

[22] Kontula O. The evolution of sex education and students' sexual knowledge in Finland in the 2000s. Sex Educ. 2010;10(4):373–386. 10.1080/14681811.2010.515095

[23] Dodd S, Widnall E, Russell AE, et al. School-based peer education interventions to improve health: a global systematic review of effectiveness. BMC Public Health. 2022;22(1):2247. 10.1186/s12889-022-14688-3. 36461024 PMC9719233

[24] He J, Wang Y, Du Z, Liao J, He N, Hao Y. Peer education for HIV prevention among high-risk groups: a systematic review and meta-analysis. BMC Infect Dis. 2020;20(1):338. 10.1186/s12879-020-05003-9. 32398032 PMC7218508

[25] Plourde KF, Ippoliti NB, Nanda G, McCarraher DR. Mentoring interventions and the impact of protective assets on the reproductive health of adolescent girls and young women. J Adolesc Health. 2017;61(2):131–139. 10.1016/j.jadohealth.2017.03.002. 28528208

[26] Temin M, Heck CJ. Close to home: evidence on the impact of community-based girl groups. Glob Health Sci Pract. 2020;8(2):300–324. 10.9745/GHSP-D-20-00015. 32606096 PMC7326521

[27] Pike C, Coakley C, Ahmed N, et al. Goals for girls: a cluster-randomized trial to investigate a school-based sexual health programme amongst female learners in South Africa. Health Educ Res. 2023;38(5):375–391. 10.1093/her/cyad025. 37405698 PMC10516375

[28] Kutywayo A, Yah CS, Naidoo NP, Malotana M, Dyani S, Mullick S. Implementing the Good Participatory Practice guidelines in the Girls Achieve Power trial in South Africa. SAGE Open. 2018;8(4):1–12. 10.1177/2158244018809149. 32983597 PMC7473095

[29] Smith P, Marcus R, Bennie T, et al. What do South African adolescents want in a sexual health service? Evidence from the South African Studies on HIV in Adolescents (SASHA) project. S Afr Med J. 2018;108(8):677–681. 10.7196/SAMJ.2018.v108i8.13013. 30182885

[30] Austrian K, Soler-Hampejsek E, Behrman JR, et al. The impact of the Adolescent Girls Empowerment Program (AGEP) on short and long term social, economic, education and fertility outcomes: a cluster randomized controlled trial in Zambia. BMC Public Health. 2020;20(1):349. 10.1186/s12889-020-08468-0. 32183783 PMC7079524

[31] Mathur S, Mishra R, Mahapatra B, Heck CJ, Okal J. Assessing layered HIV prevention programming: optimizing outcomes for adolescent girls and young women. AIDS. 2022;36(Suppl 1):S75–S83. 10.1097/QAD.0000000000003242. 35766577

[32] Shepherd J, Kavanagh J, Picot J, et al. The effectiveness and cost-effectiveness of behavioural interventions for the prevention of sexually transmitted infections in young people aged 13-19: a systematic review and economic evaluation. Health Technol Assess. 2010;14(7):1-iv. 10.3310/hta14070. 20178696

[33] South African National Department of Basic Education (NDBE); South African National Department of Health (NDOH). *Integrated School Health Policy 2012*. NDBE/NDOH; 2012. Accessed November 1, 2023. https://serve.mg.co.za/content/documents/2017/06/14/integratedschoolhealthpolicydbeanddoh.pdf

